# The Combination of Metformin and Disulfiram-Cu for Effective Radiosensitization on Glioblastoma Cells

**DOI:** 10.22074/cellj.2020.6798

**Published:** 2019-12-15

**Authors:** Narges Rezaei, Ali Neshasteh-Riz, Zohreh Mazaheri, Fereshteh Koosha, Mahmood Hoormand

**Affiliations:** 1. Radiation Biology Research Center, Iran University of Medical Sciences, Tehran, Iran; 2. Department of Radiation Sciences, School of Paramedicine, Iran University of Medical Sciences, Tehran, Iran; 3. Department of Anatomical Sciences, Medical Sciences Faculty, Tarbiat Modares University, Tehran, Iran; 4. Department of Medical Physics and Biomedical Engineering, Faculty of Medicine, Tehran University of Medical Sciences, Tehran, Iran; 5. Department of Pharmacology, Faculty of Medicine, Iran University of Medical Sciences, Tehran, Iran

**Keywords:** Apoptosis, Disulfiram, Glioblastoma, Irradiation, Metformin

## Abstract

**Objective:**

Glioblastoma (GBM) is one of the devastating types of primary brain tumors with a negligible response to standard therapy. Repurposing drugs, such as disulfiram (DSF) and metformin (Met) have shown antitumor properties in different cell lines, including GBM. In the present study, we focused on the combinatory effect of Met and DSF-Cu on the induction of apoptosis in U87-MG cells exposed to 6-MV X-ray beams.

**Materials and Methods:**

In this experimental study, the MTT assay was performed to evaluate the cytotoxicity of each drug, along with the combinatory use of both. After irradiation, the apoptotic cells were assessed using the flow cytometry, western blot, and real-time polymerase chain reaction (RT-PCR) to analyze the expression of some cell death markers such as *BAX* and *BCL-2*.

**Results:**

The synergistic application of both Met and DSF had cytotoxic impacts on the U87-MG cell line and made them sensitized to irradiation. The combinatory usage of both drugs significantly decreased the cells growth, induced apoptosis, and caused the upregulation of *BAX, P53, CASPASE-3*, and it also markedly downregulated the expression of the anti-apoptotic protein BCL-2 at the gene and protein levels.

**Conclusion:**

It seems that the synergistic application of both Met and DSF with the support of irradiation can remarkably restrict the growth of the U87-MG cell line. This may trigger apoptosis via the stimulation of the intrinsic pathway. The combinatory use of Met and DSF in the presence of irradiation could be applied for patients afflicted with GBM.

## Introduction

Glioblastoma (GBM) is a grade IV astrocytoma, regarded as one of the most aggressive and devastating cancers of the central nervous system with a dismal prognosis (1, 2). Despite the improvements in therapeutic options, treatment modalities have recently improved overall survival up to 14.6 months (3). GBM is characterized by the uncontrolled proliferation of astrocytes along with the increased rate of angiogenesis, making the disease incurable with a high recurrence rate (4, 5). Since GBM is highly resistant to recent therapies, new approaches are required to improve the treatment outcomes. Upon treatment of cancer cells with ionizing radiation and cytotoxic agents, the cell signaling pathways involved in the vital biological functions such as cell division, gene expression, and protein synthesis are effectively influenced. Ionizing radiation could cause damages to DNA and dysregulate the expression of a group of essential proteins, such as P53 and DNA-dependent kinases (6).

Radiation stimulates the expression of P53 via the posttranslational mechanism. P53 regulates the expression level of several genes and proteins that are located in the downstream of the P53 signaling pathway such as *BAX* and *P21* (7). The Bcl-2 family proteins play a significant role in the regulation of apoptosis, and they consisted of two groups of proteins, namely pro-apoptotic (BAX) and anti-apoptotic (BCL2) proteins which are capable of modulating the mitochondrial permeabilization. After irradiation, BCL2 and the caspase cascade are fully activated, and the process of apoptosis would be initiated (8).

During the last decades, molecular targeting agents, such as disulfiram (DSF) and metformin (Met), which are known as repurposing drugs, exert antitumor properties when used for the induction of cell death in different types of cancers, such as GBM (9). DSF is an Food and Drug Administration (FDA) approved drug which is a member of the carbamate family, and it has been prescribed as a safe drug for the treatment of alcohol abusers (10). Recently, researchers have highlighted DSF as an anti-cancer agent for different kinds of malignancies, including hematological cancers, breast cancer, melanoma, and especially GBM (11, 12). DSF is a safe and non-toxic compound which can easily penetrate the blood-brain barrier (BBB) (13).

Met, an FDA-approved drug, belongs to the family of biguanide agents, and it is commonly prescribed for patients who have type 2 diabetes (14). Different studies have shown that Met exerts antitumor potential against many types of tumors; however, the mechanisms underlying the anti-tumor characteristic of Met is still unknown (15-17).

Furthermore, Met could show anti-neoplastic properties via the prevention of the electron transport chain complex I (ETCI) and activation of the pathways responsible for energy homeostasis. Met is also capable of stimulating the expression of adenosine monophosphate-activated protein (AMPK) and preventing the activation of the mammalian target of rapamycin complex I (mTOR). It has been shown that Met confines the synthesis of proteins and suppresses cancer stem cells via the inhibition of the production of Foxo- 3 and AKT. Some reports indicated that Met could increase the sensitivity of tumor cells to common anti-cancer drugs such as paclitaxel and temozolomide (TMZ) (18). To date, numerous clinical trials and retrospective studies have been conducted to elucidate how Met can extend the survival of patients afflicted with cancer (19, 20). Several lines of evidence demonstrated that Met and DSF could prevent the proliferation, invasion, and metastasis of tumor cells in the pancreas (21, 22).

According to the above evidence, we hypothesized that the combination of DSF-Cu and Met with irradiation could induce apoptosis in the U87-MG cell line. Therefore, we investigated the effect of combinatory treatment with Met and DSF-Cu in the presence of radiation on the induction of apoptosis thereby the measurement of *BAX, CASPASE3, P53* and *BCL2* levels in the U87-MG cell line. Our findings could be a promising approach for the treatment of GBM patients.

## Materials and Methods

### Cell culture

In this experimental study, the U87-MG cell line was purchased from the Pasteur Institute, Tehran, Iran. Cells were cultured in high-glucose Dulbecco’s modified Eagle Medium (DMEM, Atocel, Austria) supplemented with 10% fetal bovine serum (FBS, Biowest, France) and 1% Penicillin-Streptomycin (Atocel, Austria). Cells were then incubated at 37˚C in a humidified atmosphere containing 5% CO_2_ and 95% O_2_.

This research was performed after receiving the ethics approval from the Ethics Communication of Iran University of Medical Science (IUMS, Number: 28594).

### Preparation of drugs and application of irradiation

DSF, Copper chloride (CuCl_2_), and Met (1,1-dimethyl biguanide-hydrochloride) were procured from Sigma Aldrich (Dorset-UK). DSF was then dissolved in dimethyl sulfoxide [DMSO, maximum DMSO concentration was 0.1% (v/v)]. Met and CuCl_2_ were diluted in double distilled water to reach the desired concentrations and kept at -20˚C until analysis Afterward, drugs were diluted with DMEM to adjust the required concentrations. Cells were treated with various types of drugs and then assigned into seven groups as follows: Met group; cells received only Met, Met+IR group; cells were treated with Met plus irradiation, DSF-Cu group; cells received only DSF, DSF-Cu+IR group; cells were treated with DSF-Cu along with irradiation, Met+DSF-Cu group; cells received the combination of Met and DSF-Cu, Met+DSFCu+IR; cells were treated with the combination of Met and DSF-Cu with the support of irradiation, and Control group; cells received no treatment. The 3-(4, 5-Dimethylthiazol-2- yl)-2, 5-diphenyltetrazolium bromide (MTT, Atocel, Austria) assay was applied to evaluate the cytotoxicity of the drugs. Cells were treated with various concentrations of Met (1- 15000 µM) and DSF-Cu (0.1-5 µM). Also, the impact of combinatory treatment was also examined by the MTT assay. Briefly, 24 hours after the cell-seeding, cells were pre-treated with Met for 24 hours and then treated with DSF-Cu for another 24 hours. Afterward, cells were exposed to a dose of 2Gy X-ray and incubated for 24 hours. Finally, the flow cytometry, western blot, and real-time polymerase chain reaction (RT-PCR) analyses were performed. Irradiation was carried out using a linear accelerator (Siemens, Germany) at a dose rate of 2Gy/minutes, with a field size of 40×40 cm^2^ to determine the radiosensitivity of the U87-MG cells. In accordance with treatment methods, the maximum dose of 6 MV X-ray was applied at a 1.5 cm depth of tissue. Thus, we used three layers of 0.5 cm tissue-equivalent, which were placed under the plates to ensure the electronic equilibrium. The cells were exposed to a dose of 2Gy from the posterior side of the flasks. The required dose of irradiation was calculated by the treatment planning software by which the dose of irradiation was determined as 2Gy/minutes,

### Cell survival inhibition assay

The cell viability was measured using MTT to evaluate the cytotoxicity of each drug along with the combination of both. Briefly, U87-MG cells at a concentration of 1×10^4^ were seeded on the 96-well plates and incubated at 37˚C overnight to allow the cells to adhere. The cells were then treated with Met, DSF, Cu, DSF-Cu, and the combination of both drugs. After the determination of the incubation times, cells were incubated with the MTT solution (5 mg/ml) at 37˚C for 4 hours, and then the medium was removed to solubilize the formazan crystals. Next, 100 µl DMSO was added to each well, and the absorbance was measured using an ELISA reader (Biorad laboratories, USA) at an excitation wavelength of 570 nm. The percentage of viability was calculated utilizing the comparison of the absorbance of treated cells with untreated cells. Thus, the following formula was employed to compute the percentage of viability:

Viability=treated cell absorbance/untreated cell absorbance×100.

### Flow cytometry

The Annexin-V kit was purchased from Biosciences Inc. (BD, e-Biosciences, USA). The rate of apoptosis in U87-MG cells was assessed using the Annexin-PI detection kit (BD-ebioscience) according to the manufacturer’s protocols. Briefly, 3×10^5^ cells were seeded on the 6-well plates for 24 hours and pre-treated with10 mM Met. After 24 hours, they were exposed to 1:1 µM DSF-Cu and finally irradiated with 6 MV X-Rays at the dose of 2 Gy. The cells were then harvested and then resuspended in 100 µl binding buffer. The FITC-conjugated Annexin V-PI were added and incubated for 15 minutes at room temperature. After that, the percentage of apoptotic cells was determined using the flow cytometry (BD FACS Caliber, BD Biosciences, Sanjose, CA, USA) analysis, and the obtained data were analyzed using the FlowJo software (version 7.6.1). The degree of apoptosis was detected in the FL-1 channel (green fluorescence) while necrosis was recorded in the FL-3 channel (red fluorescence). Finally, cells stained with only Annexin-V were at the early stage of apoptosis, while those double-stained with Annexin-V and PI were at the late step of the apoptosis process. Each sample had a negative control that was Annexin^-^- PI^-^ and the quadrant was set based on this sample. Samples of each group were compared with their control counterparts.

### Western blot analysis

First off, the protein contents of the cells were extracted using RIPA-buffer (Cell Signal, Germany). Next, about 20 μg of the extracted proteins (BioRad Bradford Assay, BioRad, Germany) was mixed with loading buffer (Carl Roth, Germany), and then run on sodium dodecyl sulfatepolyacrylamide gel electrophoresis (SDS-PAGE, BioRad Mini-Protean II Cell; BioRad, Germany). After the electrophoresis process, proteins were transferred onto PVDF that was located in transfer buffer containing 192 mM glycine, 10% methanol, 25 mM Tris, and pH=8.2. The membrane was blocked by 4% non-fat milk powder in phosphate-buffered saline (PBS)-0.05% Tween for 2 hours. The primary antibodies were added in blocking buffer and incubated at 4˚C overnight (1:2000 dilution, Cell Signaling Technology, Danvers, MA, USA), according to the manufacturer’s instructions. The membranes were incubated with primary antibodies against rabbit anti-BCL-2 and rabbit anti-BAX (All obtained from the Cell Signaling Technology Company). The equal protein lane loading was corroborated, utilizing a monoclonal antibody against the GAPDH protein (Sigma-Aldrich, USA). The membranes were rinsed three times with PBS-T [0.1% (v/v) Triton-X100] buffer for 30 minutes and probed with horseradish peroxidase (HRP)- conjugated secondary antibodies for 2 hours. When the washing process with PBS-T buffer was performed, the protein bands were visualized using the Odyssey Infrared Imaging System (LI-COR).

### Real time- quantitative polymerase chain reaction

The RNA isolation kit was obtained from Qiagen (USA), and the cDNA synthesis kit was purchased from Thermo Scientific (USA). In this study, total RNA was extracted from all experimental groups using Qiazol (Qiazol lysis reagent, USA), according to the manufacturer’s instructions. The integrity and concentration of the extracted RNA were determined using a Nanodrop (Thermo Scientific, USA) apparatus, by which the absorbance of the samples is read at wavelengths of 260 and 280 nm. Soon after, cDNA was synthesized using the Revert Aid™ First Strand cDNA Synthesis Kit (Thermo Scientific, MA, USA), based on the manufacturer’s recommendations. Primers were designed for GAPDH (internal control), *CASPASE3, BAX, BCL-2*, and *P53* by Pishgam Company (Iran, Tehran). Table 1 shows the sequences of primers, the accession number, and the melting temperature of primers. The suitability of the primers was confirmed using BLAST (http://blast.ncbi.nlm.gov/Blast. cgi) to identify the amplified fragment length and show that there were no non-specific binding sites on the same gene or positions of the similar sequences in other species. The relative expression of the above genes was assessed using the 2^-∆∆ct^ method for all groups. RT-qPCR was performed on an Applied Bio-System.

**Table 1:** List of primer sequences of apoptosis-related genes


Official name	Primer sequences (5ˊ-3ˊ)	Accession number	TM (˚C)

GAPDH	F: GCAGGGATGATGTTCTGG	001289745/2	86.6
	R: CTTTGGTATCGTGGAAGGAC		
P53	F: TTCCGTCTGGGCTTCTTG	001126118/1	88.1
	R: TGCTGTGACTGCTTGTAGAT		
CASPASE3	F: GGACTGTGGCATTGAGAGAG	001354779/1	81.25
	R: GGAGCCATCCTTTGAACTTC		
BAX	F: CGCCCTTTTCTACTTTGACA	001291430/1	86.61
	R: GTGACGAGGCTTGAGGAG		
BCL2	F: TGGTCTTCTTTGAGTTCGG	000633/2	86.61
	R: GGCTGTACAGTTCCACAA		


### Statistical analysis

Statistical analysis was performed using the independentsample t test and one-way analysis of variance (ANOVA) via the SPSS software version 16. The error bars represent the standard error among the different experiments. All analyses were conducted at a significance level of P<0.05. All experiments were performed in triplicate.

## Results

### Metformin and Disulfiram-Cu are individually cytotoxic to U87-MG cells and inhibit cell growth

**Fig 1:**
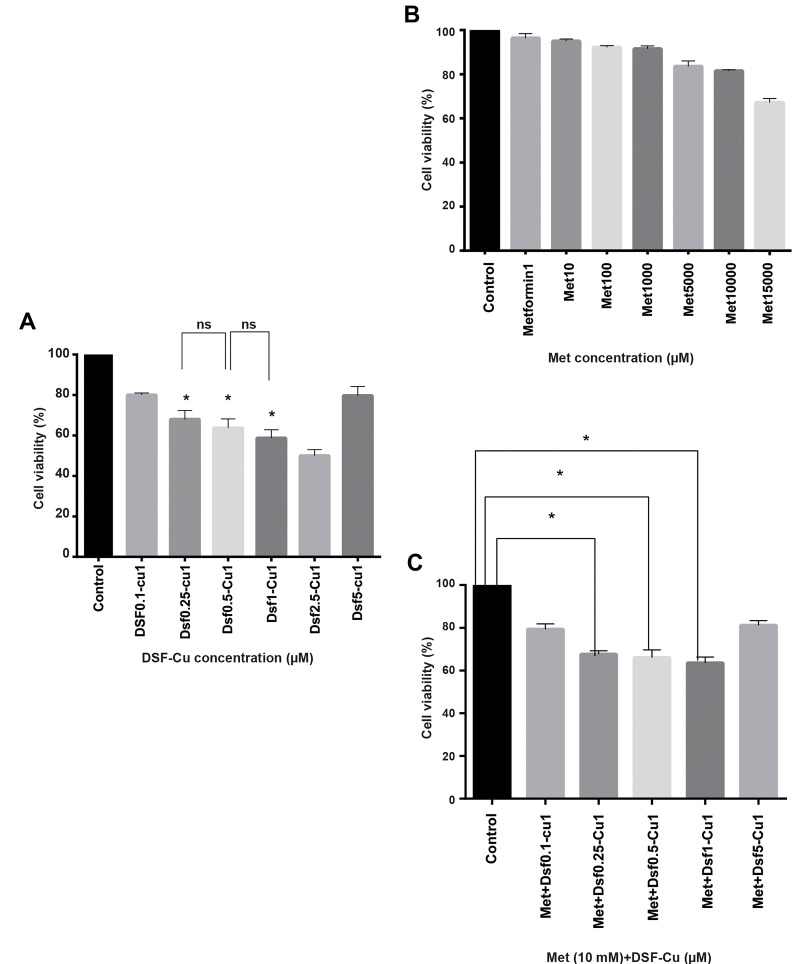
Metformin and DSF-Cu are cytotoxic to U87-MG cells when used alone and inhibit cell viability. **A.** The effect of different concentrations of DSF-Cu on U87-MG cells; DSF (0.1-5 µM), Cu 1 µM, **B.** The impact of different doses of Met (1-15000 µM) on U87-MG cells. Cells were treated and incubated with Met and DSF-Cu for 24 hours, and the MTT assay was performed at least three times, **C.** DSF-Cu increases the cytotoxic effect of Met on U87-MG cells *in vitro* in a dose-dependent manner. The MTT assay was carried out to assess the combinatory effects of Met and DSF-Cu (0.1-5 µM) and Cu (1 µM). Cells were pretreated with 10 mM Met, and after 24 hours, treated with DSF-Cu. Afterward, the MTT assay was performed 24 hours later. The error bars represent the standard error of the mean (SEM) from three repetitions for the experiments. Some error bars are too small to be seen. *; Indicates a statistically significant difference between the control and drug-treated groups at P<0.05 vs. the control group, DSF; Disulfiram, Met; Metformin, and ns; Nonsignificant.

At first, the MTT assay was performed to evaluate the effect of Met and DSF-Cu on U87-MG cell viability. Cells were exposed to different concentrations of DSF, Cu (0.1-5µM) and Met (0.1-15000 µM). Treatment with DSF or Cu alone had no significant effect on cell viability. Interestingly, as shown in Figure 1A, the combination of DSF-Cu with different concentrations decreased cell viability (P<0.05). The cytotoxicity of DSF was dependent on Cu. Met significantly reduced the cell viability in a dose-dependent manner (Fig .1B).

### Disulfiram-Cu enhanced the cytotoxicity effect of metformin on U87-MG cells in a dose-dependent manner

To assess the cytotoxicity of Met in combination with drugs that are activated in reducing environments first, cells were pre-treated with 10 mM Met for 24 hours and then treated with different concentrations of DSF along with 1 µM Cu for 24 hours. Finally, the MTT assay was conducted to assess the toxicity of each compound or the combination of them. Figure 1C shows that there was no significant difference among 0.25, 0.5 and 1 µM DSF when combined with 1 µM Cu (P>0.05), and the highest toxicity was observed in equimolar (1:1) ratio of DSF to Cu. Furthermore, the morphology of the cells changed as they became more rounded-shape. Thus, junctions between the cells were not observed.

### Disulfiram-Cu and metformin as well as their combination, induced apoptosis in U87-MG cells

**Fig 2:**
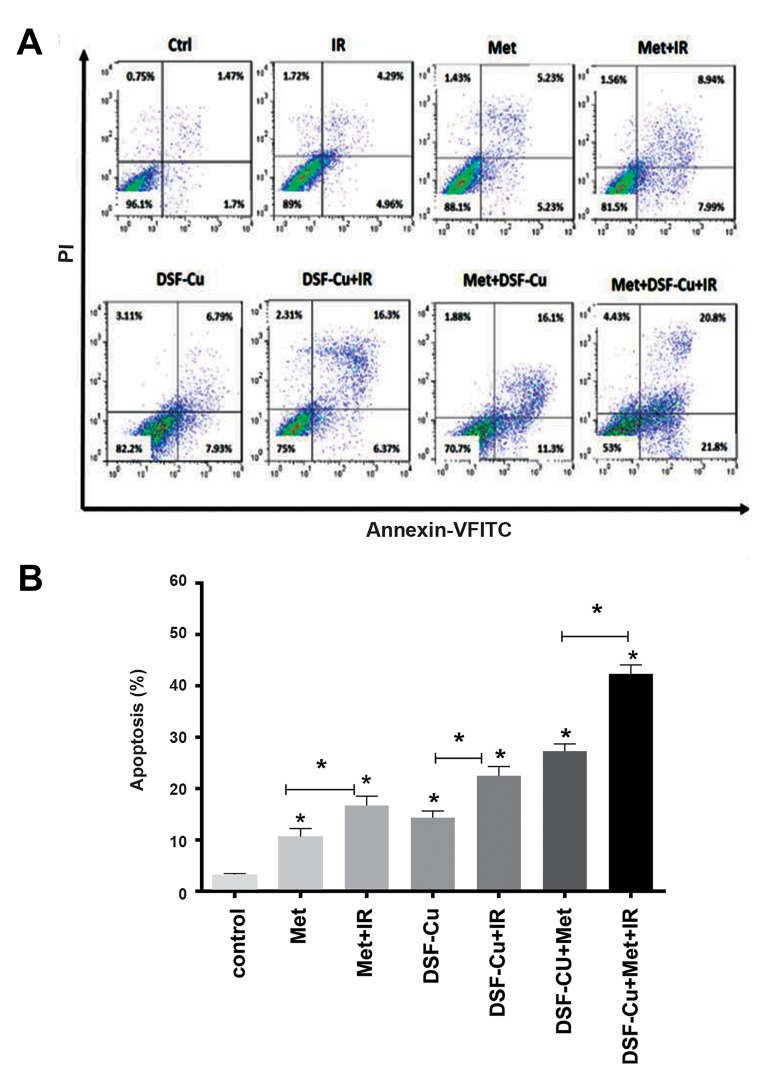
DSF-Cu, Met, and the combination of both promote apoptosis in U87-MG cells. Cells were treated with 10 mM Met at a ratio of 1:1 µM DSF-Cu and irradiation at a dose of 2 Gy. Apoptosis assay was performed 24 hours after the treatment with drugs and IR exposure. **A.** The flow cytometry plots show the early and late apoptosis in the treated groups and **B.** Total apoptosis in different treatment groups. Data are expressed as the mean ± SEM deduced from experiments performed in triplicate, *; P<0.05 versus the control group, DSF; Disulfiram, Met; Metformin, and IR; Irradiation.

Apoptosis was evaluated in cells treated with Met, DSFCu, and a combination of both drugs. For this aim, cells were exposed to 10 mM Met, and DSF-Cu at a ratio of 1:1 µM for 24 hours. The combinatory effect of both drugs was assessed with Annexin/PI staining that is measured by the flow cytometry analysis. As shown in Figure 2A, a single treatment with Met or DSF-Cu induced cell death in U87-MG cells. As compared with the control or single treatment, the percentage of apoptosis was significantly increased when the synergistic usage of both drugs was applied (P<0.05). In cells treated with Met, the percentage of apoptosis was 10.62 ± 1.60% (P<0.05), whereas the rate of apoptosis was 14.34 ± 1.29% in cells treated with DSF-Cu (P<0.05). Also, the percentage of cell death was 27.31 ± 1.37% when the combined treatment strategy was used (P<0.05).

### Radiation enhances apoptosis in U87- MG cells

The radiosensitization effect of Met and DSF-Cu was determined when they were used alone or in combination with each other on cells was in the presence of 6 MV X-ray at a dose of 2 Gy. After 24 hours of irradiation, the rate of apoptosis was measured in cells. As compared with the control group and the groups treated with single drugs, apoptosis was significantly (P<0.05) increased in the Met+IR group (16.72 ± 1.79%). The percentage of cell death was 22.48 ± 1.79% (P<0.05) in the DSF-Cu+IR group whereas the rate of apoptosis was reported 42.35 ± 1.73% in the Met+DSF-Cu+IR group. The radiosensitization effect was more pronounced (P<0.05) in the Met+DSF-Cu+IR group when compared with other treatment groups. As a whole, the synergistic role of Met and DSF-Cu considerably inhibited the rate of cell growth in U87-MG cells. Besides, the combination of both drugs promoted irradiation mediated cytotoxicity (Fig .2A, B).

### Metformin, Disulfiram-Cu, and the combination of drugs reduced BCL2 protein levels and increased BAX levels in U87-MG cells

**Fig 3:**
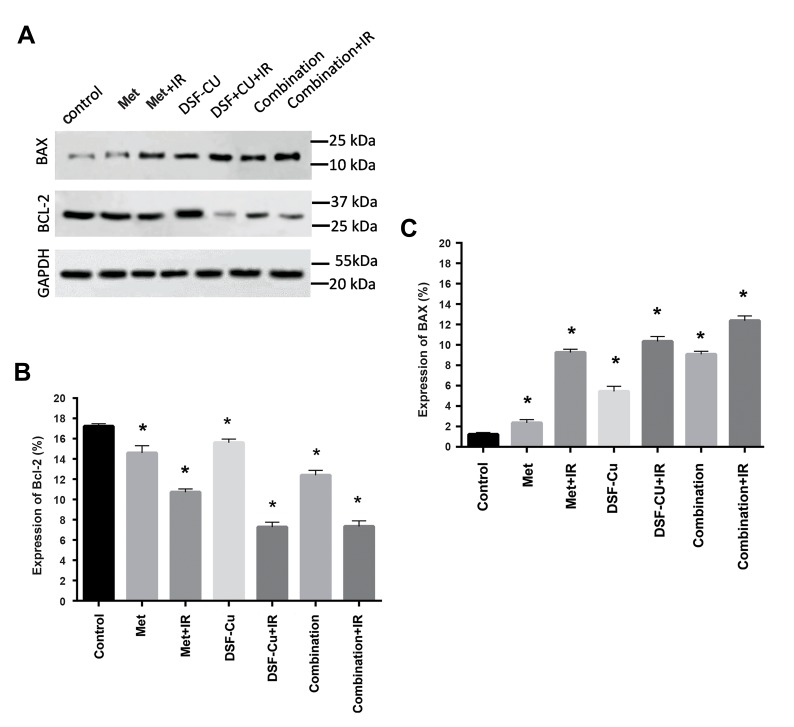
Met, DSF-Cu, and the combination of both suppress BCL- 2 protein levels and increase BAX levels in U87-MG cells. **A.** The expression levels of BCL-2 and BAX proteins were measured in U87-MG cells by the western blot analysis 24 hours after treatment with 10 mM Met at a ratio of 1:1 µM DSF-Cu and 2Gy IR, **B.** Western blot of BAX, BCL-2 with and GAPDH. BCL-2 expression was significantly decreased, and **C.** While the expression of BAX was markedly increased in all treatment groups. *; P<0.05 vs. the control group, Met; Metformin, DSF; Disulfiram, and IR; Irradiation.

To investigate the mechanisms underlying the apoptotic role of DSF-Cu, Met, and the combination of both, the levels of apoptosis-related proteins, such as BCL2 and BAX were assessed by the western blot analysis 24 hours after the treatment periods and irradiation (Fig .3A). The western blot assaydemonstrated that the expression of BCL2 was markedly (P<0.05) decreased when cells treated with drugs alone or in combination with each other (Fig .3B). Also, the levels of BAX was significantly (P<0.05) increased between each treatment group and the control group (Fig .3C). The increase in the expression of Bax and the reduction in the expression of BCL 2 were remarkable (P<0.05, Met: 0.002, Met+IR: 0.020, DSF-Cu: 0.004, DSF-Cu+IR: 0.020, Combination: 0.018, Combination+IR: 0.035) higher in the cells treated with the combination of both drugs with the support of irradiation in comparison with other treatment groups.

### Metformin Disulfiram-Cu and the combination of both with irradiation regulate anti-apoptotic and pro-apoptotic markers in U87-MG cells

**Fig 4:**
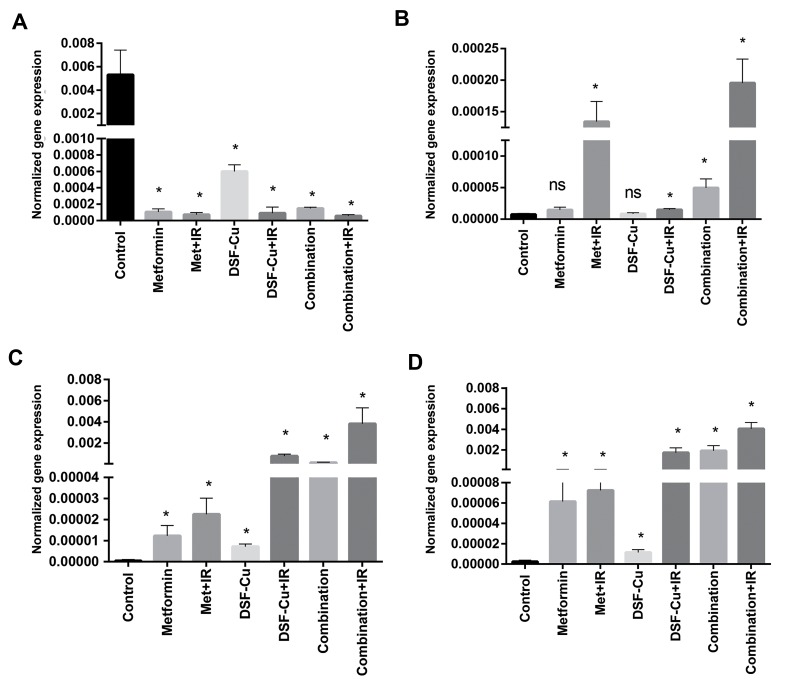
Regulation of anti-apoptotic and pro-apoptotic genes. U87-MG cells were treated with 10 mM Met at a ratio of 1:1 µM DSF-Cu, and the combination of both drugs in the presence/absence of irradiation at a dose of 2 Gy. Then, RT-qPCR was performed to evaluate the expression levels of apoptosis-related genes. **A.** The relative expression level of *BCL-2*, **B.** The relative expression of *BAX*, **C.** The relative expression of *CASPASE-3*, and D. The relative expression of *P53*, 24 hours after the incubation times. The internal control was *GAPDH*, and the relative gene expression was compared with the untreated control group. Some error bars are too small to be detected. Data are expressed as the mean ± SEM. *; P<0.05 versus the control group, Met; Metformin, DSF; Disulfiram, ns; Non significant, and RT-qPCR; Real time-quantitative polymerase chain reaction.

The gene expression levels of anti-apoptotic and pro-apoptotic genes that regulate the intrinsic pathway of apoptosis were evaluated using the RT-PCR method. The incubation of U87-MG cells with Met, DSF-Cu, and the combination of both drugs caused the overexpression of *BAX, P53*, and *CASPASE3*, but decreased the expression of *BCL2*, as compared with the control or untreated group. The expression of *BCL2* was significantly (P<0.05) diminished in all groups as depicted in Figure 4A. *BAX* expression was considerably increased between the treatment groups and the control group (P<0.05). However, in cells treated with Met, the expression of *BAX* remained unchanged (P>0.05) as displayed in Figure 4B. Figure 4C shows that, as compared with the control group, the expression of *CASPASE3* significantly substantially elevated in all treated groups (P<0.05). As shown in Figure 4D, the expression of *P53*, as compared with the control group, was significantly increased in all treated groups (P<0.05).

## Discussion

In recent decades, studies dealing with repurposing drugs, such as DSF and Met possessing the anti-tumor properties are rapidly increasing with the hope of seeking new strategies to fight cancer cells (9). The available treatment strategies have a poor prognosis for the cure of glioblastoma, which is a lethal brain cancer in humans; therefore; new treatment modalities for the therapy of GBM are warranted. The application of drugs that target the metabolism of cells in combination with conventional treatment may yield satisfactory results (23, 24). Based on the results of the present study, the combination of Met and DSF-Cu with the support of radiation had cytotoxic effects on U87-MG cells in which significantly decreased proliferation and increased rate of apoptosis was observed in cells. In other words, the combination of Met and DSF-Cu in the presence of irradiation was more effective in the suppression of cell proliferation and promotion of apoptosis than the single use of radiation, Met or DSFCu. Jivan et al. (25) showed that Met in combination with DSF-Cu had cytotoxic effects on oesophageal squamous cell carcinoma (Oscc) and Met empowered the cytotoxicity of DSF-Cu.

Additionally, Met augmented Cu transport into Oscc cells and caused a substantial increase in the number of apoptotic cells and cell death (25). We have also demonstrated that DSF and Cu had no cytotoxic impact on U87-MG cells when used alone, but the synergistic use of both drugs dramatically decreased cell growth and viability even in the presence of 1 µM DSF. Above this concentration, the viability of cells was increased up to 5 µM.

Some previous studies indicated the biphasic cytotoxic effect of DSF on U87-MG cells in which the cells underwent apoptosis when the compound was used at the concentrations of 0.25, 0.5, and 1 µM. The concentrations above 10 µM did not show any signs of cytotoxicity (22, 26). Several lines of evidence implicated that repurposing drugs, such as Met and DSF-Cu are capable of preventing cell growth, arresting cell cycle progression, as well as triggering autophagy and apoptosis via intrinsic pathway when employed individually in tumor cells especially in GBM (17, 27). Met and DSF-Cu can penetrate the BBB without causing any significant side effects; thus exhibiting low cytotoxicity even when combined with irradiation to act as radiosensitizers (28, 29).

A large body of *in vivo* and *in vitro* research demonstrated that Met and DSF-Cu could increase the potency of chemotherapeutic agents and radiotherapy when added to the therapeutic regimen as the addition of these two drugs are capable of elevating the levels of* BAX, P53, CASPASE-3*, as well as decreasing the level of *BCL-2* in some types of malignancies such as pancreas, lung, and breast cancers (30-32). The precise mechanism(s) by which Met and DSF-Cu exert anticancer potential is still opaque. It is thought that DSF could prevent the activation of the proteasome, as well as the expression/activation of aldehyde dehydrogenase (ALDH) and nuclear factor B (NFkB). Besides, these effects, when DSF is combined with Cu can serve to stimulate the production of reactive oxygen species (ROS) (33, 34). In a study performed by Haji et al. (35), they reported that DSF could incite the initiation of cell death pathway by the overexpression of *P21* and *BAX* genes within pancreatic cancer cells. Correspondingly, Feng et al. (36) showed that treatment of GBM cells with DSF-Cu increased the levels of Bax and Caspase-3, and also decreased the Bcl-2 level.

Thus, it seems that apoptosis is mediated via the intrinsic pathway in response to the exposure of the cells to DSF-Cu (36). Sesen et al. (29) indicated that Met at a dose of 300 mg/kg did not induce any significant cell toxicity when administered to the mice *in vivo*. Of note, the administration of Met at a dose prescribed for diabetic patients is not the allowed drug dosage; rather, the lowest side effects were observed when administrated for patients with diabetes. In the case of malignancies, the concentration of Met could be increased up to 10mM as we employed this dose in our experiments (14). In one distinct investigation conducted in Iran, it has been shown that the administration of Met altered the expression of caspase-8, -9, and *PARP-1* in breast cancer cells; however, the expression of *CASPASE-3* remained unchanged (37). Also, different studies have demonstrated that treatment of pancreatic and breast cancer cells with Met, induced apoptosis via the intrinsic pathway in which the expression of BAX was elevated while BCL-2 expression was proteins diminished (30, 32). Apoptosis regulates the proliferation of cells (38), and the Bcl-2 family proteins play a vital role in the modulation of apoptosis in different cells (8). When the regulation of the BCL2 family proteins is impaired, cytochrome c is released from the mitochondria, leading to the activation of a caspase cascade in which some caspases such as CASPASE-9 and -3 are fully activated and can initiate the process of cell death (39). Moreover, P53, when present in the cytoplasm, regulates the permeability of mitochondrial membrane as a pro-apoptotic protein which causes the release of cytochrome c and induces caspase cascade activation which is required for cell death (7). According to our the flow cytometry analyses of the present study, the combinatory treatment significantly induced apoptosis in U87-MG cells as approved by the western blot results, showing a significant reduction in the expression of the B-2 and an increase in proapoptotic proteins such as BAX. Different death inducing and survival genes contribute to the control of the apoptosis process, and these genes are capable of regulating via internal or external signals (40). It is apparent that the combination of Met and DSF-Cu can induce apoptosis, led to the overexpression of BAX and P53, as well as the activation of CASPASE3 and decreasing the expression of BCL2. At the same time, in the presence of irradiation, these effects were amplified, and in comparison with the combinatory treatment, we can conclude that the combination of both drugs with irradiation can act as a radiosensitizer and induce apoptosis through the intrinsic pathway. The contradictory results obtained from other investigations may be due to discrepancies in the experimental conditions, diverse cell origin, and/ or cell function and, empirical method models that need complementary investigations. It seems that the synergistic use of both drugs effectively stimulated the apoptotic pathways within the cells; however, we cannot rule out the involvement of other death pathways such as autophagy as they share common protein when activated in response to external and internal insults. Further studies are required to illuminate whether the induction of apoptosis is not the sole pathway for the efficacy of the combinatory treatment with Met and DSF-Cu.

## Conclusion

Our findings showed that the combined treatment with Met and DSF-Cu with the support of radiation could be an advantageous method for the prevention of U87-MG cells growth in comparison with treatment with Met, DSFCu or irradiation alone. The molecular mechanism of action can be related to the changes in the expression level of proteins and genes involved in the intrinsic pathway of apoptosis. This study is the first preclinical evidence evaluating the combined impact of Met and DSF-Cu with the aid of radiation on the growth of U87- MG cells as this approach increased the sensitivity of U87-MG cells to radiation. Altogether, these findings provide hope for the cure of GBM patients who are resistant to conventional therapies. Further investigations should be carried out to determine the precise mechanism of action and therapeutic potency of the combination of Met and DSF-Cu.
